# Supplementary material for: Probabilistic photonic computing with chaotic light
Source: Nat Commun. 2024 Dec 1;15:10445. doi: 10.1038/s41467-024-54931-6 (PMC11609296; doi:10.1038/s41467-024-54931-6)
Supplement: Supplementary file 1 — Supplementary Information [file 41467_2024_54931_MOESM1_ESM.pdf]

Supplementary Information for
Probabilistic Photonic Computing with Chaotic Light

Frank Brückerhoff-Plückelmann[1], Hendrik Borras[2], Bernhard Klein[2], Akhil Varri[1], Marlon Becker[3,4], Jelle Dijkstra[5], Martin Brückerhoff[6], C. David Wright[7], Martin Salinga[8], Harish Bhaskaran[9], Benjamin Risse[3,4], Holger Fröning[2], Wolfram Pernice[1,5]*

[1]Physical Institute, University of Münster; Münster, 48149, Germany.
[2]Institute of Computer Engineering, University of Heidelberg; Heidelberg, 69120, Germany.
[3]Institute for Geoinformatics, University of Münster; Münster, 48149, Germany.
[4]Faculty of Mathematics & Computer Science, University of Münster; Münster, 48149, Germany
[5]Kirchhoff-Institute for Physics, University of Heidelberg; Heidelberg, 69120, Germany.
[6]DEVK RE; Cologne, 50668, Germany.
[7]Department of Engineering, University of Exeter; Exeter, EX44QF, UK.
[8]Institute of Materials Physics, University of Münster; Münster, 48149, Germany.
[9]Department of Materials, University of Oxford; Oxford, OX43PJ, UK.

*Correspondence to: wolfram.pernice@kip.uni-heidelberg.de

SUPPLEMENTARY METHODS

Chaotic Light as a Tunable Random Number Generator

Chaotic light, for example from an amplified spontaneous emission (ASE) source, can be described as the superposition of many oscillating electric fields of different frequencies with random phase and random intensity:

$$E(t) = \sum_{k=-\infty}^{k=\infty} (x_k + iy_k) \cdot e^{i(\omega_0 + k\Delta w)} + c.c. \qquad (1)$$

Here x_k and y_k are both zero mean gaussian distributed random variables with a variance proportional to the spectral power density[1]. The interference between all frequency components causes a time varying field intensity. Thus, the number of incident photons n measured at a detector within a measurement interval follows a M-fold Bose-Einstein distribution[2–4]. The degeneracy factor M depends on the ratio between the coherence time τ_c of the optical field and the measurement time T [2]. In the case of large mean photon numbers n_{mean} obtained within a measurement interval T, the probability density function of the M-fold Bose Einstein distribution can be simplified to:

$$P(n, n_{\mathrm{mean}}, M) = \frac{M^M}{n_{\mathrm{mean}}\Gamma(M)} \cdot \left(\frac{n}{n_{\mathrm{mean}}}\right)^{M-1} \cdot \exp\left(-\frac{M \cdot n}{n_{\mathrm{mean}}}\right) \qquad (2)$$

The degeneracy factor M, which corresponds to the number of independent temporal coherence cells of the ASE within the measurement interval, asymptotically approaches T/τ_c in the limit $T \gg \tau_c$ *(28)*. Overall, the photon number fluctuations lead to a second noise source apart from the shot noise which is proportional to the squared mean photon number:

$$\mathrm{Var}(n) = n_{\mathrm{mean}} + \frac{n_{\mathrm{mean}}^2}{M} \qquad (3)$$

Supplementary Fig. 2a shows the relative variance of the photon number distribution. For the simulation, we assume a chaotic light field with a rectangular spectral power density that has a width of 200 GHz. The relative variance decreases for increasing measurement times as the number of independent temporal coherence cells within the measurement interval increases. We fit a Bose-Einstein to the photon count statistics in Supplementary Fig. 2b for a measurement interval of 50 ps. The fit converges to a degeneracy factor of M = 10.64

comparable to the asymptotic approximation of M = 10. Most importantly, the variance is proportional to the squared mean photon number and thus the relative noise level does not approach zero for large photon numbers as for the shot noise term. Thus, we can modulate the noise level by modulating the mean photon number.

Since the number of photons within a time interval is directly linked to the optical intensity, we can use this effect to also modulate the signal to noise ratio. In the following, we model our detection system by a low pass filter with rectangular shape and a cutoff frequency of 30 GHz. Supplementary Fig. 3a shows the simulated intensity profile for a chaotic light source discarding the terms oscillating with the frequency of the wave (approximately 193 THz). The traces contain various frequencies components given by the beating between the spectral components of the broadband light source. In case the optical bandwidth is smaller than the cutoff frequency of the detector, the detector can resolve all frequency components, corresponding to a voltage trace proportional to the intensity. In the other case, the detector acts as a low pass filter and removes the higher order components in the intensity trace, effectively smoothing the signal as shown in Supplementary Fig. 3b. Therefore, the signal to noise ratio depends on the ratio between the optical and electrical bandwidth. It remains unity for optical bandwidths smaller than the detector cutoff and increases for larger bandwidths due to the smoothening of the lowpass filter as shown in Supplementary Fig. 3c. Setting the ratio between the optical and electrical bandwidth allows to set the signal to noise ratio of the system even in the case of large photon numbers and intensities. Finally, we encode our symbols in the mean intensity of the chaotic light source, see Fig. 2a in the main text. To ensure that two symbols are uncorrelated, the time between them must be larger than the correlation time of the chaotic source itself. In the case that the optical bandwidth is smaller than the electrical one, the autocorrelation of the output voltage is given by the second order degree of coherence of the chaotic light field and is 2 for zero time-lag. In case that the optical bandwidth exceeds the electrical one, the autocorrelation for zero time-lag decreases and the characteristic time scale is given by the electrical bandwidth of the system. In our case of 200 GHz optical bandwidth and an electrical bandwidth of around 30 GHz, the time between two symbols of 56.8 ps ensures that they are uncorrelated.

Interference of Chaotic Light

The superposition of two independent chaotic light source populating the same optical frequencies behaves like a single chaotic light source since the sum of two independent zero

mean gaussian random variables is again a zero mean gaussian random variable. As the variances add up, the intensities of the fields are also summed up. To avoid deploying several chaotic light sources, we deploy delay lines instead as sketched in Supplementary Fig. 4a. The inference between the various frequency components will strongly impact the measured output distribution if the delay time is short in comparison to the coherence time of the chaotic field as we practically implement a Mach-Zehnder interferometer. However, the interference of the field with its delayed copy behaves like the sum of two independent fields for large delay times. We simulate the detector signal for our system with an optical bandwidth of 200 GHz and an electrical bandwidth of 30 GHz. Supplementary Fig. 4b shows the mean detector voltage depending on the delay time. The output voltage follows the first order degree of coherence and approaches 0.5 for delay times larger than the coherence time, effectively adding up the intensities in both arms. Since the Mach-Zehnder configuration acts like a spectral filter and hence impacts the optical bandwidth, the SNR at the detector strongly depends on the delay time for small delays as shown in Supplementary Fig. 4c. However, for large delays, the SNR approaches the one of a single chaotic light source again. We compare the probability density function in the cases of no delay, a delay in the order of the coherence length and a delay much longer than the coherence time. For a short delay, the PDF has a different shape than a single chaotic light source but recovers the initial form for larger delay times. The PDF can also be nonzero for negative voltages even though the intensity is inherently positive, since the voltage is proportional to the low pass filtered intensity.

System Probability Density Function

Apart from noise contributions arising from the chaotic light and electronic ground noise, system imperfections impact the final measured voltage distribution. First, the transimpedance amplifier of the photodetectors do saturate for large input powers, effectively reducing the width of the distribution especially for large mean voltages as shown in Fig. 2 of the main manuscript. We model the saturated signal $f(x)$ of an input x with a saturation parameter a_{sat} :

$$f(x) = a_{\mathrm{sat}} \cdot \left(1 - \mathrm{e}^{-\frac{x}{a_{\mathrm{sat}}}}\right) \qquad (4)$$

Since $f(x)$ is a differentiable, strictly monotonically increasing function, $f^{-1}(y)$ exists and the probability density function $\tilde{p}(y)$ of the saturated output satisfies:

$$\tilde{p}(y) = \frac{p\left(f^{-1}(y)\right)}{f'\left(f^{-1}(y)\right)} \qquad (5)$$

Here $p(x)$ is the probability density function of the input, in this case the M-Bose-Einstein distribution of the optical intensity, see Supplementary Equation (2). For a measured mean of $\tilde{P}(y, y_{\text{mean}})$, the mean of $P(x, x_{\text{mean}})$ is:

$$x_{\text{mean}}(y_{\text{mean}}) = \frac{M \cdot a_{\text{sat}}}{(1 - y_{\text{mean}}/a_{\text{sat}})^{1/M}} - M \cdot a_{\text{sat}} \qquad (6)$$

Moreover, there is a limited extinction ratio from the modulators and additional ASE injected after modulation from the optical amplifiers. Consequently, light is present at the detector even if the symbol “0” is send. Both effects will shift the Bose-Einstein distribution by an amount y_{DC}. Combining both effects, the optical contribution to the measured noise distribution is:

$$\tilde{P}(y, y_{\text{mean}}) = \frac{P(f^{-1}(y + y_{\text{DC}}), x_{\text{mean}}(y_{\text{mean}} + y_{\text{DC}}))}{f'\left(f^{-1}(y + y_{\text{DC}})\right)} \qquad (7)$$

Finally, we convolve the probability density distribution with the one of a zero mean Gaussian with standard deviation σ_{el} to also include the electronic noise as described in the main text. We fit the standard deviation of $\tilde{P}$ to the measured one shown in Fig. 2. For the fit parameter we find $\sigma_{\text{el}} = 0.053$, $M = 3.046$, $a_{\text{sat}} = 2.684$ and $y_{\text{DC}} = 0.011$. All “model” plots shown in main text deploy these fit parameters.

Bayesian Neural Network Tooling

Both Bayesian Neural Networks (BNNs) presented in this work were built with the same software stack. The probabilistic programming language Pyro[5] is used as the primary tool for designing the BNNs. For quantization and discretization of values to certain bit widths the quantization aware training library Brevitas is utilized. Pyro and Brevitas both are built on top of PyTorch[6], a widely used machine learning library for Python[7]. To speed up numerical computations for integrating the photonic PDF shown in Supplementary Equation (7) we employ numba[8], a just-in-time compiler for Python code. Finally, for hyperparameter searches, experiment tracking and to interface with our local compute infrastructure we use the experiment management library seml.

Gaussian Bayesian Neural Network

The network follows the overall design shown in Fig. 5a of the main text. Instead of the Sigmoid activation function used in the original LeNet-5 architecture this network uses rectified linear units (ReLU) as activation functions. To accelerate the off-chip training procedure, we only approximate the photonic system with a Gaussian BNN. Supplementary Fig. 6a shows how the probabilistic average pooling layer is implemented in Pyro. As the intensity of the chaotic light is positive by definition and the electronic digital to analog interface has a limited precision (maximum 8bit due to the arbitrary waveform generator), we choose a QuantReLU activation function. In this way, the input is mapped to an output activation between 0 and 1 with a 4bit uniform quantization. Apart from mapping the inputs to the correct range and deploying a reasonable number of bits for analog computing, we approximate the probabilistic sampling process. Instead of the complex probability distribution shown in Supplementary Equation (7), we use Gaussian distributions. Furthermore, we approximate the dependency between standard deviation and mean for a single symbol by $\sigma(x) = \sigma_{ground} + \sigma_{slope} \cdot x$ with $\sigma_{\text{ground}} = 0.052$ and $\sigma_{\text{slope}} = 0.42$. Deploying the encoding scheme shown in Fig. 2f of the main text, the standard deviation of a distribution with mean μ can be tuned between

$$\sigma_{\text{min}}(\mu) = \frac{N}{\sqrt{N}} \cdot \left(\frac{\mu}{N} \cdot \sigma_{\text{slope}} + \sigma_{\text{ground}}\right) \qquad (8)$$

$$\sigma_{\text{max}}(\mu) = \sqrt{\left(\frac{N-1}{\sqrt{N-1}} \cdot \sigma_{\text{ground}}\right)^2 + \left(\mu \cdot \sigma_{\text{slope}} + \sigma_{\text{ground}}\right)^2} \qquad (9)$$

With μ the mean signal of the photo diode, N=9 the number of uncorrelated symbols per distribution, and σ_{ground} and σ_{slope} fit values for the gaussian noise approximation, as observed with the hardware.

The BNN is then trained using the SVI implementation of Pyro with trace_ELBO as the loss function. We then use seml to find good hyperparameters, with the final settings being:

- Optimizer: ADAM
- Learning rate: 0.003162

- Batch size: 256
- Pyro guide: AutoDelta
- Pyro training and inference algorithm: SVI
- Loss: trace_ELBO
- Training samples: 10
- Initialization of noise levels (l in Fig. S6): 1.0
- Number of epochs: 225
- Seed of all involved random number generators at the start of the experiment: 42
- Compute device: CPU

All other parameters were left at their defaults, where applicable. In general, we find that the model shows good convergence over a wide range of learning rates, noise level initializations and for different optimizers. Of note is that while convergence with a low noise level initialization is good, a large initialization afterwards results in a more diverse distribution of noise levels and widens the final MI distribution for OOD samples shown in Fig. 5e. For the final model we find a wide final distribution of noise levels as shown in Supplementary Fig. 6c, in particular for the first average pooling layer.

Photonic Bayesian Neural Network

The photonic BNN adapts the parameters learned using the Gaussian BNN and is utilized to verify that the BNN still performs within expectation, when executed on the actual hardware. Schematically the implementation is shown in Supplementary Fig. 6b. Notably this implementation utilizes the full pipeline of generating patterns for the arbitrary waveform generator (AWG), stochastically rounding to hardware precision, average pooling within the photonic crossbar array and finally sampling of individual light pulses from the actual photonic PDF shown in Supplementary Equation (7).

In the following the individual steps shown in Supplementary Fig. 6b are explained in detail:

- QuantReLU: Adapts the quantization from the gaussian BNN and ensures numerical compatibility.

- AWG pattern generation: Here the individual light pulse patterns for the arbitrary waveform generator employed in the photonic hardware are computed. The computation is dependent on the input from the previous layer (μ) and the noise levels (L) learned by the gaussian BNN and the number of symbols per distribution ($N = 9$). Where the final pattern $\mathbf{x}(\mu)$, see Supplementary Equation (12), can be described as follows using vector calculus:

$$\mathbf{x}_{\min}(\mu) = \left(\frac{\mu}{N}, \dots, \frac{\mu}{N}\right) \quad (10)$$

$$\mathbf{x}_{\max}(\mu) = (\mu, 0, \dots, 0) \quad (11)$$

$$\mathbf{x}(\mu) = \mathbf{x}_{\min}(\mu) \cdot (1 - L) + \mathbf{x}_{\max}(\mu) \cdot L \quad (12)$$

- Stochastic Quant: Here the AWG patterns $\mathbf{x}(\mu)$ are rounded to four bit, using a standard stochastic rounding scheme, where the probability of rounding up or down depends on the distance of the value to be rounded to the nearest discrete level. This effectively reduces the bias from accumulating rounding errors, as would usually be the case with standard, fixed rounding.

- Avg. Pool: Standard PyTorch average pooling is applied, which approximates the photonic crossbar array. Due to the previous quantization only a deterministic number of result values (means) can exist afterwards, however the computation incurs floating-point errors, which makes static pre-computation of the photonic PDFs difficult.

- Batched PDF sampling: This operation dynamically requests the computation of new photonic PDFs, as new mean values appear, while suppressing rounding errors and dynamically caching existing PDFs, to reduce runtime overhead. Sampling from a precomputed PDF is then accomplished by resampling from the cumulative distribution function using uniformly distributed samples.

- Summation of samples: The samples drawn for each light pulse are summed together to form the final result. This result is then passed on to the following deterministic layers of the network.

SUPPLEMENTARY DISCUSSION

Speed Considerations

Several characteristics contribute to the throughput and latency of the processor. The group velocity of 150.000 km/s for the employed silicon nitride waveguides at the wavelength of 1550 nm imposes a lower limit on the latency. For a crossbar array with size on the mm to cm scale, the minimal latency time is on the order of 6 to 60 ps, solely due to the propagation time of the light pulse. The symbol rate of the electronic interface and the multi-symbol encoding of the distribution have a larger impact on the latency time. With 9 subsequent symbols needed and a symbol rate of 17.6 GBaud for one channel, the total latency time for the full probabilistic computation is on the order of 550 ps. Parallel sampling does not decrease the latency of a single computation, but increases the throughput, as several samples are required to gather the output statistics of the BNN. As highlighted in the Supplementary Fig. 7, around 100 samples from the output distribution are required for a reliable out of domain detection. Considering four wavelength channels for parallel sampling, it takes 25 x 0.55 ns = 13.75 ns to obtain those samples. This time could be reduced by using more channels in the C-Band, where the ASE source emits, for parallel sampling

SUPPLEMENTARY REFERENCES

1. Vannucci, G. & Teich, M. C. Computer simulation of superposed coherent and chaotic radiation. *Appl. Opt.* **19**, 548 (1980).

2. Goodman, J. *Statistical optics*. (2000).

3. Shimoda, K., Takahasi, H. & Townes, C. H. Fluctuations in Amplification of Quanta with Application to Maser Amplifiers.pdf. *J. Phys. Soc. Japan* **12**, 686–700 (1957).

4. Pietralunga, S. M., Martelli, P. & Martinelli, M. Photon statistics of amplified spontaneous emission in a dense wavelength-division multiplexing regime. *Opt. Lett.* **28**, 152 (2003).

5. Bingham, E., Chen, J. P., Szerlip, P. & Goodman, N. D. Pyro : Deep Universal Probabilistic Programming. 0–5.

6. Schrijvers, T., Van Den Berg, B. & Riguzzi, F. Automatic Differentiation in Prolog. *Theory Pract. Log. Program.* 1–4 (2023) doi:10.1017/S1471068423000145.

7. Rossum, G. Van & Drake, F. L. Python Reference Manual. *October* **22**, 9117–9129 (2006).

8. Lam, S. K., Pitrou, A. & Seibert, S. Numba: A LLVM-based Python JIT Compiler. in *Proceedings of LLVM-HPC 2015: 2nd Workshop on the LLVM Compiler Infrastructure in HPC - Held in conjunction with SC 2015: The International Conference for High Performance Computing, Networking, Storage and Analysis* (2015). doi:10.1145/2833157.2833162.

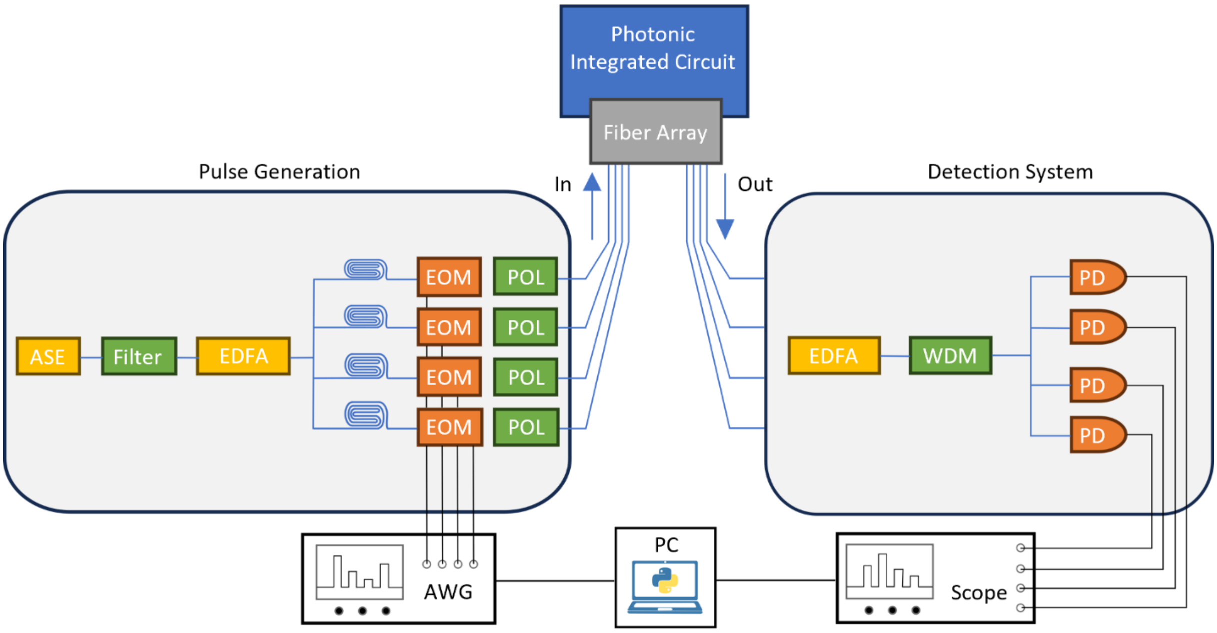

Supplementary Figure 1. **Experimental setup for driving the probabilistic processor.** We split the chaotic light into four arms and delay them with respect to each other before shaping the pulse forms with an electro-optic modulator. Before coupling to the photonic crossbar array, we adjust the polarization of each pulse shape independently. We amplify the output of the integrated photonic circuit upon spectral sampling.

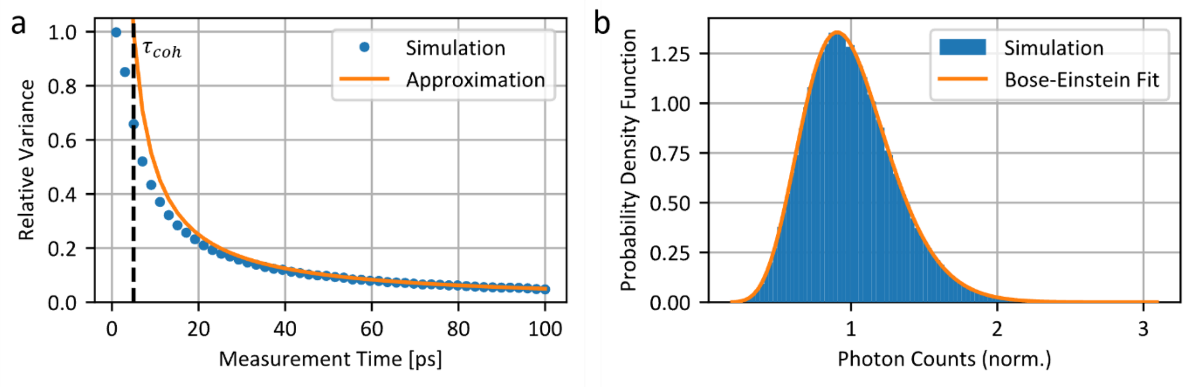

Supplementary Figure 2. **Photon count statistics of a chaotic light source with 5 ps coherence time. a** The relative variance of the number of photons measured within a time interval depends on the ratio between the measurement time and the coherence time of the light. It decreases for an increasing measurement time as the fluctuations average each other out. **b** The probability density function for a measurement time of 50 ps follows a M-fold Bose Einstein distribution with degeneracy factor M=10.64.

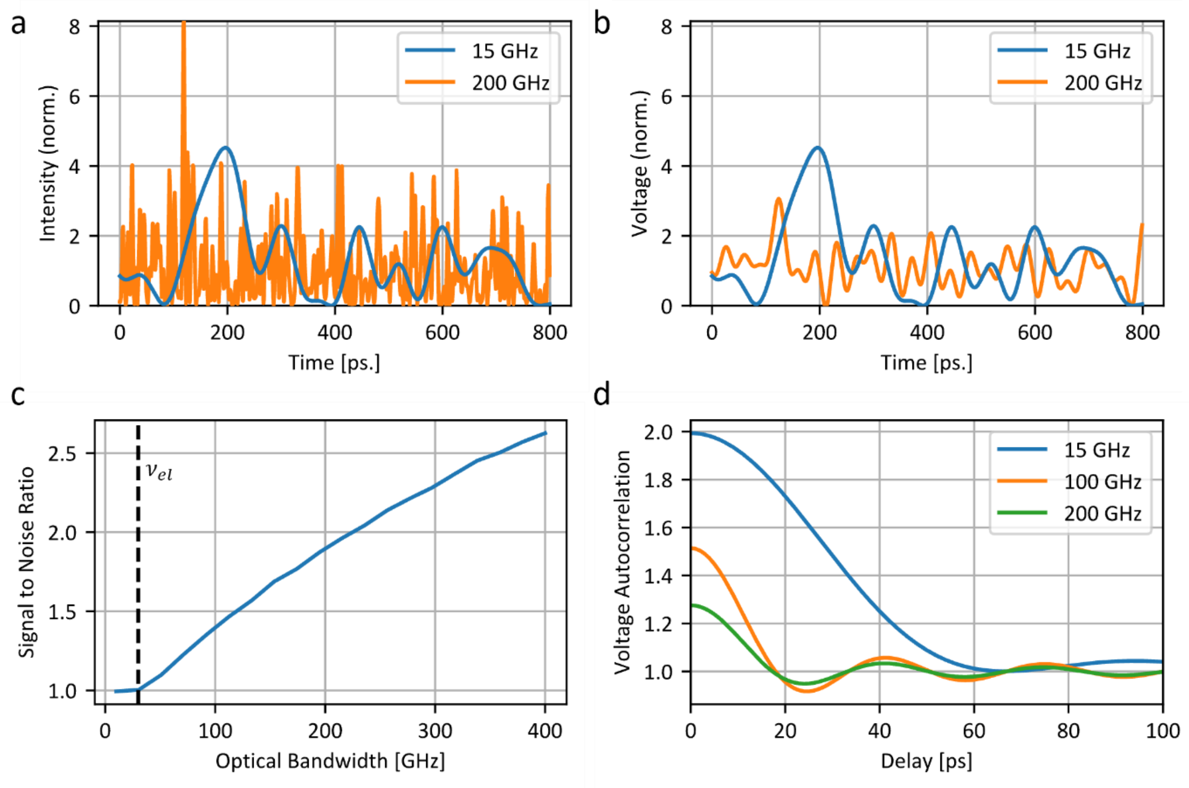

Supplementary Figure 3. **Measuring chaotic light with a bandwidth limited electrical system. a** The random beating between the various frequency components of the chaotic light leads to fluctuations in the intensity of the field. The time scale of the fluctuation's scales inversely to the bandwidth of the chaotic light. **b** We simulate the detector voltage trace for the chaotic light field with an electrical readout circuit limited to a bandwidth of 30 GHz. The lowpass filter removes potential high frequency components in the optical intensity profile, effectively smoothening the measured signal for large optical bandwidth. **c** We simulate the detector response for various optical bandwidths of the chaotic light to obtain the mean (signal) and the standard deviation (noise) of the voltage trace in dependence on the optical bandwidth. For an optical bandwidth below electronic one all fluctuations are resolved, and the SNR is 1 whereas it decreases for larger optical bandwidth due to the lowpass filtering effect of the detector. **d** The autocorrelation of the detector voltage depends on the optical and electrical bandwidth. In case the electrical system can fully resolve the intensity fluctuations, the correlation time is inversely proportional to the optical bandwidth, otherwise it depends on the electrical bandwidth.

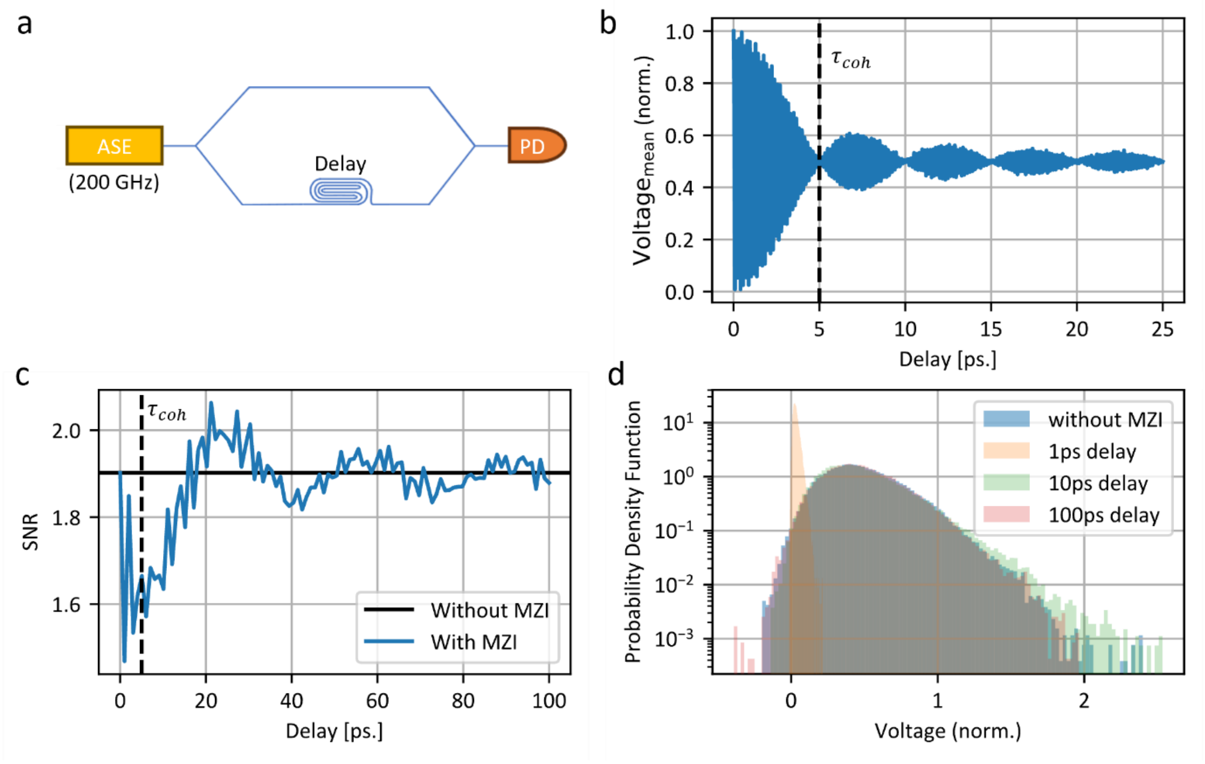

Supplementary Figure 4. **Interference of chaotic light fields.** **a** We deploy delay lines to effectively create independent copies from a single chaotic light source instead of using several physical independent chaotic sources. The structure effectively implements a Mach-Zehnder interferometer (MZI). **c** For delay times much larger than the coherence length, interference effects are averaging out and the output intensity is the sum of the input intensities. **b** For large delay times also the SNR recovers to the value of a single chaotic light source as the impact of the effective spectral filter decreases. **d** Overall, the probability density function is the one of a single chaotic source for large delay times.

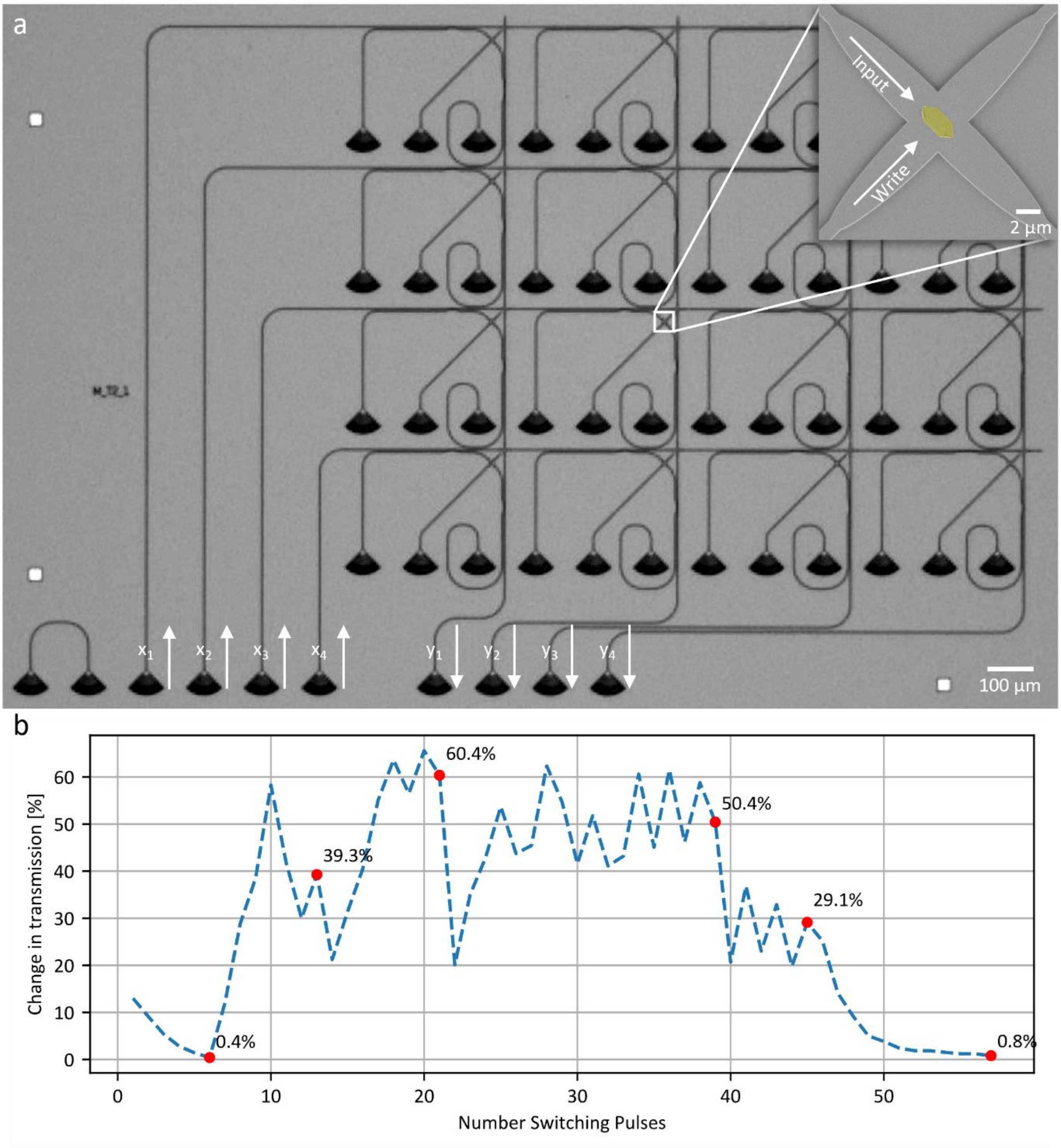

Supplementary Figure 5. **Computing with phase change materials. a** We integrate Germanium-Antimony-Telluride (GST) cells into the photonic circuit as tunable non-volatile attenuators. For the photonic crossbar array, each matrix element is represented by one crossbar array cell which consists of directional couplers, a crossing with the GST and couplers to switch the GST. **b** By sending high power optical pulses through the GST cell we can increase the transmission by amorphizing the GST and decrease the transmission by crystallizing. We can program the transmission through the GST with an error smaller than 1%.

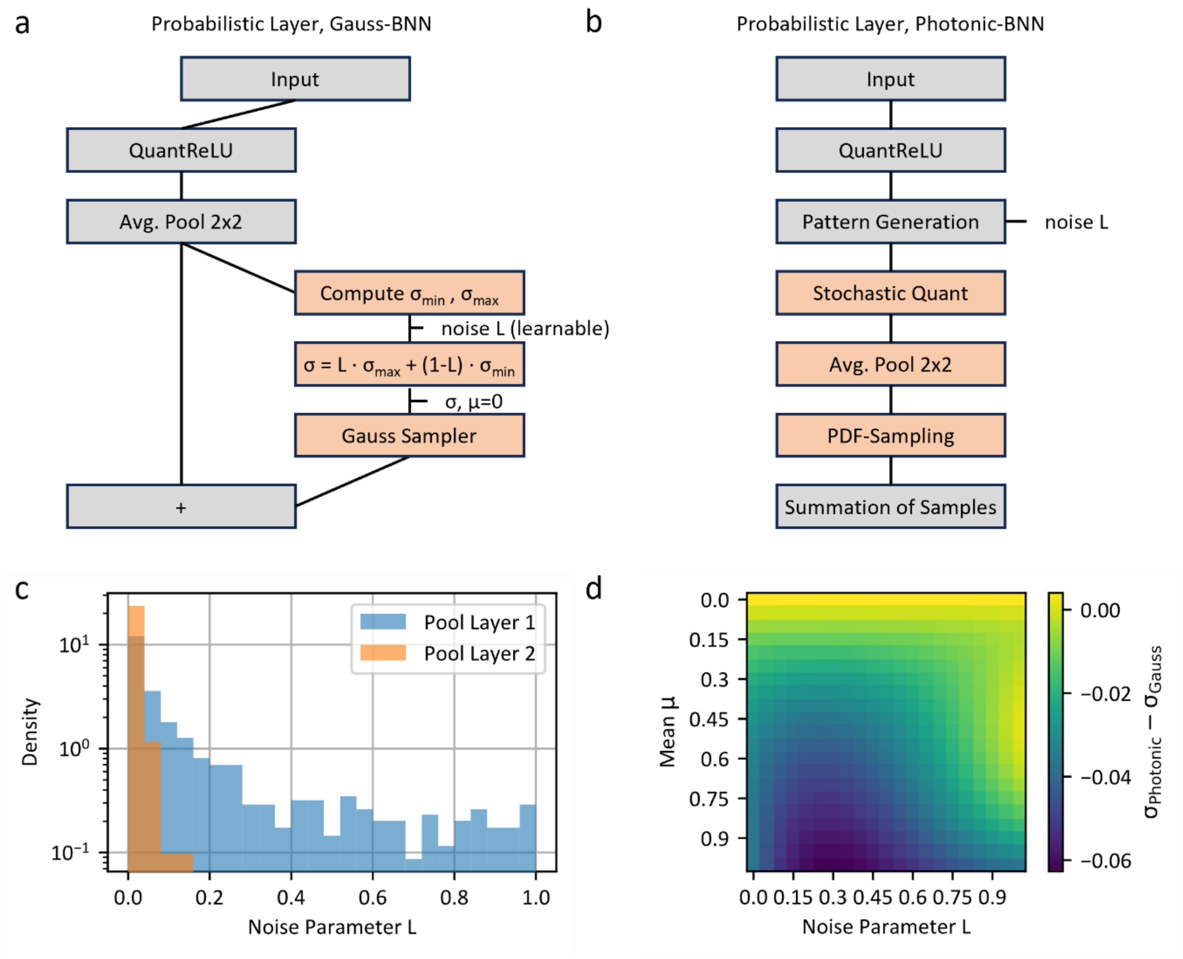

Supplementary Figure 6. **Bayesian neural network model. a** Schematic of the probabilistic average pooling layer for the Gaussian Bayesian neural network (BNN). Here the photonic probability density function (PDF) with the encoding of Fig. 2f is approximated as a single gaussian distribution. **b** Schematic of the probabilistic average pooling layer for the photonic BNN. Here the photonic PDF is computed exactly, along with hardware imperfections being represented by the stochastic quantization. The model also takes care of effectively caching pre-computed PDFs, thus drastically reducing the computational overhead. Furthermore, we also effectively suppress floating point rounding errors, to further improve computational performance. Note that this model introduces no new parameters, when compared to the Gaussian BNN, and is used as a direct drop-in replacement thereof. **c** Final distribution of noise levels for the two probabilistic average pooling layers of the developed Gauss BNN, based on LeNet-5. It is of note that the noise levels are per-design clamped between 0 and 1 and that the first layer appears to have a much more use for significant noise levels, compared to the second. **d** Difference in the standard deviation of the programmed noise levels when transferring the model parameters from the Gauss BNN to the Photonic BNN. The maximal absolute difference

in the standard deviation is ca. 0.06 which does not impact the overall performance of then BNN as shown in the main text.

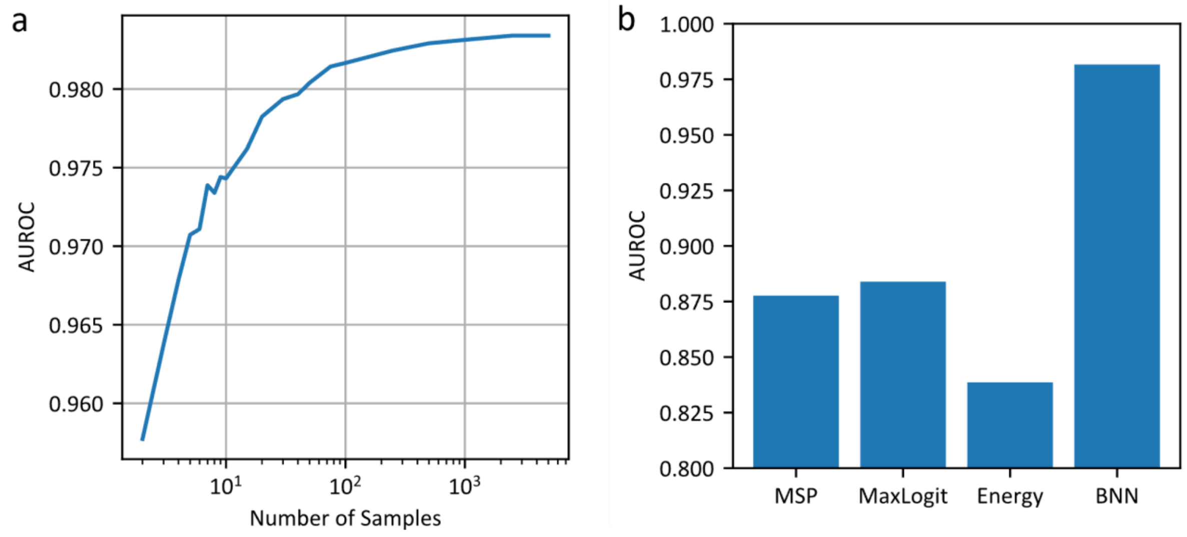

Supplementary Figure 7. **OOD detection performance. a** The area under receiver operating characteristic (AUROC) for out-of-domain (OOD) rejection is shown in dependence on the number of samples drawn from the output distribution. The AUROC describes the performance of the OOD classifiers to detect outlier samples. For an ideal OOD detection the AUROC would be 1. **b** Comparison of OOD detection performance between the presented BNN and deterministic approaches. The deterministic approaches are based on an equivalent ANN, trained with Cross-Entropy Loss and otherwise the same hyperparameters. All deterministic methods base their OOD Detection on the output of the ANN. The BNN readily outperforms the ANN based approaches, due to its more complete statistical nature and higher methodical complexity.

Total Samples per Second (N/s)	(4.554 ± 0.101) x 1e+06
AWG pattern generation (N/s)	(1.230 ± 0.065) x 1e+08
Stochastic Quantization (N/s)	(1.188 ± 0.031) x 1e+07
Avg. Pooling (N/s)	(2.243 ± 0.226) x 1e+09
Batched PDF sampling (N/s)	(7.905 ± 0.189) x 1e+06
Sample summation (N/s)	(3.961 ± 0.353) x 1e+09

Supplementary Table 1. **Sampling rates for different operations on a CPU.** We benchmark the performance of the photonic probabilistic processor by performing identical operations on a CPU, 2x AMD EPYC ROME, 32 Cores / 64 Threads, and measuring the sampling rate for each operation. The CPU deploys a Mersenne Twister for (pseudo) random number generation and we perform the experiment for N=1990656 samples. While the deterministic CPU system performs deterministic operations as pooling also at GS/s rates, probabilistic operations impose a bottleneck and slow down the full operation to MS/s rates.
