# Supplementary material for: Probabilistic photonic computing with chaotic light
Source: Nat Commun. 2024 Dec 1;15:10445. doi: 10.1038/s41467-024-54931-6 (PMC11609296; doi:10.1038/s41467-024-54931-6)
Supplement: Supplementary file 2 — Transparent Peer Review file [file 41467_2024_54931_MOESM2_ESM.pdf]

Peer Review File

Probabilistic Photonic Computing with Chaotic Light

Corresponding Author: Professor Wolfram Pernice

This file contains all reviewer reports in order by version, followed by all author rebuttals in order by version.

Version 0:

Reviewer comments:

Reviewer #1

(Remarks to the Author)
Review

In this manuscript, the authors propose a way to perform probability distribution calculation of random variables with a physical noise source (or, as they call, entropy source). The effort builds on the research group's previous work on matrix-vector multiplication via WDM and photonic crossbar arrays with phase-change memory. To my understanding, here they propose adapting the same hardware to instead manipulate "chaotic light" to independently control the mean and variance of output probability distributions and use WDM to enable "single shot" readout of such probability distributions. I would like to salute the authors' effort to comprehensively explain the physics of their system in detail and with a high level of clarity, both in the main text and in the Supplementary materials. Although I have reservations about the practical benefits of such approach, to me the idea sounds interesting and novel from an academic standpoint. I will recommend to the editor acceptance with minor revisions. Below I will enumerate a few points that I felt needed improvement.

1. One of the main motivations of this study is to mitigate the "major challenges for conventional deterministic hardware." The authors claim that "Monte Carlo methods are employed to provide approximate solutions," but do not introduce a sanity benchmark for the fraction of computational load MC takes relative to the overall training process. In addition, the authors need to do a more comprehensive job of explaining the advantages of using true physical randomness over digital processing with pseudorandom number generators. It is not obvious to me. Finally, since the authors already used conventional electronic hardware to train their BNN, it would be appropriate to explain the speedup in computation that could be achieved by using the photonic processor.
2. A point of confusion is where the (controlled) noise comes from. Does it come from the chaotic nature of light? Or simply the fact that electronic noise is independent and identically distributed (iid)? Statements like "The noise level is solely encoded in the waveform modulated on the carrier signal." (page 8, line 236) are confusing to me. The authors explain in detail how they manipulate the variance relative to the mean, but I don't understand intuitively why this was designed in such a way. Is this mechanism made so complicated in order to achieve single-shot readout? It seems to be that post-processing multiple readouts would have been sufficient to manipulate the PDF in various complex ways.
3. Re: Naïve rejection of OOD images. The authors show the successful rejection of the OOD '9' image via mutual information. While statistics is an interesting added dimension that traditional ANNs don't have, the same image would have been equally rejected by the output layer by simply computing the cross entropy loss in the output layer. The authors need to compare using mutual information from the output of the BNN against using crossentropy loss. If they are equivalent, MNIST is simply not a good use case of Bayesian NNs. If that's the case, could the authors enumerate a few machine learning tasks that are uniquely suited for Bayesian NNs, where OOD detection is both critical and not achievable by deterministic networks? As an off-topic suggestion, I would like to point the authors to look for applications in the cryptography domain, e.g. physical unclonable functions or optical steganography.
4. Limit to scalability explained in the discussion. What limits the number of channels that can be used for the same wavelength channel? Can you explain the tradeoff between coherence time, optical filter bandwidth and sampling rate?

Minor Questions
- In Extended Data Fig. 3, what is meant by signal to noise ratio? How is it measured? The result is not intuitive to me. Also, how is the optical bandwidth experimentally manipulated as shown in Extended Data Fig. 3c? Is this just a simulation?
- In page 7, line 210, the authors write "In contrast, the standard deviation is not linearly decreased because the electronic ground noise of the readout circuit is independent from the optical intensity." What is the source of electronic ground noise? Is this an intended behavior? In other words, does this scheme rely on the electronic ground noise (thermal noise?) to

decouple mean from standard deviation? I thought the main mechanism was the chaotic light interference as explained in Eq. S3. Please clarify.
- On page 8, line 226, the authors write “As in the deterministic (convolutional) counterpart 35, we encode the convolution kernel weights in the GST cells and encode the image pixel values in the mean input intensities.” In convolutional kernels, the kernel component for different colors/channels is different. In this architecture, the GST-based weights cause the kernel component to be color-insensitive. This draws a fundamental limitation of the convolution capability. Can you clarify what it would take to use this hardware for multi-channel/multi-dimensional kernels?
- Typo in Fig. 5a “kernal”
- Typo in Supp. Fig. 6a “probabilsitc”
- Typo in Bayesian Neural Network tooling section, “hyperparamter”

Reviewer #2

(Remarks to the Author)
- Intro

In recent years, analog computing has experienced a resurgence thanks to the latest achievements in deep learning (DL). This research topic is of great interest, with efforts being made by both academic researchers and prominent companies such as IBM, NVIDIA, and Google. With the enormous potential of DL no longer under discussion, the effort is now on finding new ways to deploy these AI models efficiently in terms of computing speed, throughput, and, most importantly, power consumption. Analog photonics, in particular, has already demonstrated its ability to implement a plethora of DL models, from fully connected networks, to spiking neural networks and even reservoir computing.
Analog devices for AI fall into two main categories: (I) neuromorphic processors, which emulate the architecture and operating principles of DL, and (II) AI co-processor accelerators, which aim to improve a specific part of the DL workflow. The latter tipically aim to improve matrix multiplications, as they constitute the most demanding workload in DL inference.
In this paper, the authors report a method for developing a neuromorphic processor capable of implementing a Bayesian neural network (BNN). Their device relies on amplified spontaneous emission to implement a chaotic light (an ideal entropy source). Having a broad optical bandwidth, this chaotic light can be used in a photonic crossbar array in wavelength division multiplexing (WDM). The crossbars are based on phase-change materials acting on the amplitude of light as tuneable weighting elements.

- Comments on the manuscript

In the manuscript the authors propose an interesting method to implement a Bayesian neural network (BNN) with photonics. Instead of trying to implement a more “standard” network such as a multilayer perceptron, where noise limits the scalability and resolution of photonic implementations, they focus on an architecture where photonic noise is a resource rather than a burden. From another perspective, instead of trying to somehow replicate digital electronics, their device aims to enable a novel functionality (the implementation of a BNN) that is beyond the reach of its electronics counterpart. The BNN is implemented in photonics through a chaotic light source, electro absorption modulators (EAM) used to impress inputs in time amplitude modulation, a photonic crossbar array with phase change materials (PCM) as the WDM weighting matrix, and photodiodes attached to wavelength demultiplexers.
The authors provide a convincing discussion of why relying on WDM photonics for probabilistic computing overcomes the shortcomings of electronic counterparts (high speed, broadband optical sources, avoidance of sequential sampling, use of a single source).The authors provide a convincing discussion regarding why relying on WDM photonics for probabilistic computing overcomes the shortcomings in the electronic counterparts (high speed, broadband optical sources, avoidance of sequential sampling, use of a single source).
The manuscript provides sufficient information on the probabilistic encoding of inputs (i.e., how both the mean and the variance of input distributions can be modulated) and how the PCMs are used within the photonic crossbar to perform multiplications acting on the amplitude of optical signals.
The combination of the main and supplementary material provides sufficient information for the reproducibility of the work. Images are well composed, with appropriate captions, and of a sufficiently high resolution. The English is generally good, with a few typos (e.g., “in fast and efficient way” at page 4).

However, the manuscript has some shortcomings that should be addressed to improve its quality, and the understanding of the potential impact of the presented research.
The manuscript lacks a state-of-the-art comparison with other photonic probabilistic processors. Some references to some other works are provided, but it lacks a proper comparison that would allow to assess the degree of novelty introduced by this work and its potential impact.
A discussion of the scalability and possible integration of such a device would improve the quality of the paper. In this regard, the authors state “[…] employing chaotic light as a carrier leads to interesting scaling properties.” but this is developed in a non-quantitative way with “Note that in our experiments the number of parallel samples was mainly limited by the number of input channels of the oscilloscope […]”. To how many input channels could the structure be scaled? Presumably, the photonic crossbar array may be impractical to be implemented when increasing the number of inputs, since its dimension scales quadratically. Moreover, do the authors envisage a way to integrate the device? A semiconductor optical amplifier can be used as the chaotic light source, but programming the PCMs may become a challenge if everything is integrated (?).
While the first implementation of a novel device is almost always interesting in itself, a section discussing its shortcomings

and possible turnarounds would be impactful. The paper has the potential to become a reference for the field of probabilistic photonic computing.
Regarding the speed of the device, the authors report an overall sampling rate of about 70 GSa/s. However, while the sampling rate is given, the authors do not outline the speed of their processor in terms of throughput and actual input generation rate (sequential pulses are used to shape input distributions). For example, a processor based on a Mach-Zehnder mesh has a throughput limited by the light crossing time of the device. As for how a BNN works, the output is a series of statistics. It is not clear from the paper whether extracting such statistics requires only a forward pass or not. Furthermore, the photonic probabilistic processor is only used in 2 out of 7 layers within the developed BNN (other layers are deterministic). How does this affect the computation of the final layers of the BNN, which are run on a digital computer? Do these need to be run several times as the output of the probabilistic layers are distributions? The authors could reasonably respond to this comment by saying that the answer should be obvious if you know how BNN works. However, providing some insight into this would allow the paper to be understood by a wider audience.

- Summary

The authors propose a novel and interesting solution for implementing a probabilistic photonic processor. The potential of this work may be significant, but a proper comparison with the state of the art is required to assess its actual degree of novelty. Addressing the issues raised in this review should improve the quality of the work and potentially make it suitable for publication in Nature Communications.

Version 1:

Reviewer comments:

Reviewer #1

(Remarks to the Author)
In a previous review, I had recommended the editor to accept this for publication after providing minor revisions. In this iteration, the authors have satisfactorily addressed my revision suggestions. I appreciate the insight into how deterministic approaches are inferior in rejecting OOD samples. I still suspect that relatively small optimizations in deterministic hardware or software algorithms would be sufficient to improve probabilistic neural networks performance. As this field develops, it would be great to gain insight into what fundamentally can only be achieved via a physical entropy source and hardware-assisted PDF evaluation. It is understood that this is, however, outside the scope of this article.

Reviewer #2

(Remarks to the Author)
The authors have carefully addressed all the issues raised in my previous review. The overall quality of the paper has improved significantly. In my opinion, the paper is more reader-friendly. It has the potential to have a good impact, which is paramount.

I confirm all my positive comments on the previous review and hope to see more efforts from the scientific community in photonic probabilistic computing in the future. As I said, I particularly appreciated the use of optical noise as a resource rather than a burden.

I think the paper is now ready for publication in Nature Communications.

I thank the authors for all their responses. As a side, scientific consideration, I must disagree with a statement in the authors' response regarding scalability. The authors say that "photonic crossbar arrays in general suffer from scaling problems compared to their electronic counterparts". If we consider memristor crossbar arrays as the electronic counterpart, their scalability is also severely limited by parasitic effects. In this case, photonics has a better perspective, as in principle even long interconnections can be supported. The technological advances in fabrication processes will tell us how much we can push in scalability.

Open Access This Peer Review File is licensed under a Creative Commons Attribution 4.0 International License, which permits use, sharing, adaptation, distribution and reproduction in any medium or format, as long as you give appropriate credit to the original author(s) and the source, provide a link to the Creative Commons license, and indicate if changes were made.
In cases where reviewers are anonymous, credit should be given to 'Anonymous Referee' and the source.
The images or other third party material in this Peer Review File are included in the article's Creative Commons license, unless indicated otherwise in a credit line to the material. If material is not included in the article's Creative Commons license and your intended use is not permitted by statutory regulation or exceeds the permitted use, you will need to obtain permission directly from the copyright holder.
To view a copy of this license, visit https://creativecommons.org/licenses/by/4.0/

Response Letter Probabilistic Photonic Computing with Chaotic Light

Frank Brückerhoff-Plückelmann, Hendrik Borras, Bernhard Klein, Akhil Varri, Marlon Becker, Jelle Dijkstra, Martin Brückerhoff, C. David Wright, Martin Salinga, Harish Bhaskaran, Benjamin Risse, Holger Fröning, Wolfram Pernice

Response to reviewers

We thank both referees for their insightful reviews of our manuscript and their very helpful recommendations on how to improve it. We have followed these recommendations in full, as described in detail below.

Reviewer #1:

In this manuscript, the authors propose a way to perform probability distribution calculation of random variables with a physical noise source (or, as they call, entropy source). The effort builds on the research group's previous work on matrix-vector multiplication via WDM and photonic crossbar arrays with phase-change memory. To my understanding, here they propose adapting the same hardware to instead manipulate "chaotic light" to independently control the mean and variance of output probability distributions and use WDM to enable "single shot" readout of such probability distributions. I would like to salute the authors' effort to comprehensively explain the physics of their system in detail and with a high level of clarity, both in the main text and in the Supplementary materials. Although I have reservations about the practical benefits of such approach, to me the idea sounds interesting and novel from an academic standpoint. I will recommend to the editor acceptance with minor revisions. Below I will enumerate a few points that I felt needed improvement.

Our Response:

We thank the referee for her/his positive feedback on our work and especially the technical questions regarding the comparison to more conventional hardware and software implementations. We have added additional information and discussion to the manuscript as described in the following at the corresponding comments.

1. One of the main motivations of this study is to mitigate the "major challenges for conventional deterministic hardware." The authors claim that "Monte Carlo methods are employed to provide approximate solutions," but do not introduce a sanity benchmark for the fraction of computational load MC takes relative to the overall training process. In addition, the authors need to do a more comprehensive job of explaining the advantages of using true physical randomness over digital processing with pseudorandom number generators. It is not obvious to me. Finally, since the authors already used conventional electronic hardware to train their BNN, it would be appropriate to explain the speedup in computation that could be achieved by using the photonic processor.

Our Response:

We thank the reviewer for raising this discussion. The time to generate random numbers with pseudorandom number generators (PNRGs) strongly depends on the quality of PRNG and the hardware used. We base our experiments with conventional hardware on PyTorch, which uses a Mersenne Twister for (pseudo) random number generation. This provides a good balance between speed and statistical randomness, making it suitable for many applications and is the standard implementation for PyTorch's CPU backend.

We benchmarked the photonic BNN, see Extended Data Figure 6b, with conventional hardware and tracked the computational workload of each step:

Total Samples per Second (N/s)	(4.554 ± 0.101) x 1e+06
AWG pattern generation (N/s)	(1.230 ± 0.065) x 1e+08
Stochastic Quantization (N/s)	(1.188 ± 0.031) x 1e+07
Avg. Pooling (N/s)	(2.243 ± 0.226) x 1e+09
Batched PDF sampling (N/s)	(7.905 ± 0.189) x 1e+06
Sample summation (N/s)	(3.961 ± 0.353) x 1e+09

Table: Sampling rates for different operations on a CPU. Evaluated for N=1990656 samples.

Here the photonic PDF is already precomputed and thus the workload is only due to sampling from a PDF, not computing the shape of the PDF. As one can see, both probabilistic operations, the stochastic quantization to simulate the DAC and the batched PDF sampling both are around 3 orders of magnitude slower than the deterministic operations. This is also the reason, why all digital computation were performed on 2x AMD EPYC ROME, 32 Cores / 64 Threads and not on GPUs, as the probabilistic sampling, to be more precise, the sequential operations and large state associated with PRNG, and not the matrix multiplications are the bottleneck for the system. In comparison to the MS/s rates for the conventional systems the photonic processor is not slowed down by the probabilistic sampling and thus can operate at GS/s rates (as the deterministic photonic counterpart).

We have added the workload comparison to the Extended Data and have included a paragraph in the discussion of the main manuscript.

2. A point of confusion is where the (controlled) noise comes from. Does it come from the chaotic nature of light? Or simply the fact that electronic noise is independent and identically distributed (iid)? Statements like “The noise level is solely encoded in the waveform modulated on the carrier signal.” (page 8, line 236) are confusing to me. The authors explain in detail how they manipulate the variance relative to the mean, but I don’t understand intuitively why this was designed in such a way. Is this mechanism made so complicated in order to achieve single-shot readout? It seems to be that post-processing multiple readouts would have been sufficient to manipulate the PDF in various complex ways.

Our Response:

The reviewer is correct that the (controlled) noise only arises from the chaotic nature of the light. The intensity noise is directly proportional to the intensity and is also proportional the square root of the ratio between bandwidth of the electronic readout and optical bandwidth of the chaotic carrier. As modulating the electronic and optical bandwidth in GHz rates is more challenging from an engineering viewpoint (and might drastically deteriorate the pulse shape), we chose to modulate only the intensity. However, this links the variance directly to the mean. Thus, we employed multi-symbol encoding to overcome this issue. It would indeed be possible to obtain a similar behavior by postprocessing several readouts or only performing photonic probabilistic sampling and computing the MVM on deterministic hardware[1]. However, our operation scheme is closest to the “default” photonic crossbar arrays and especially does not require any communication between the sender and receiver about the temporal length of the encoding which is fixed. Thus, further integration will be easier as the interface and data flow can be used as for deterministic photonic crossbar arrays.

We have added this clarification to the discussion of the measurement data.

3. Re: Naïve rejection of OOD images. The authors show the successful rejection of the OOD ‘9’ image via mutual information. While statistics is an interesting added dimension that traditional ANNs don’t

have, the same image would have been equally rejected by the output layer by simply computing the cross entropy loss in the output layer. The authors need to compare using mutual information from the output of the BNN against using crossentropy loss. If they are equivalent, MNIST is simply not a good use case of Bayesian NNs. If that's the case, could the authors enumerate a few machine learning tasks that are uniquely suited for Bayesian NNs, where OOD detection is both critical and not achievable by deterministic networks? As an off-topic suggestion, I would like to point the authors to look for applications in the cryptography domain, e.g. physical unclonable functions or optical steganography.

Our Response:

While the Cross-Entropy Loss is a suitable loss function to train an artificial neural network for classification tasks like MNIST, it is not applicable for uncertainty estimation during inference as the expected/"correct" probability/"class-label" is not known during inference. The reviewer is correct, that there are deterministic ways for uncertainty estimation after training the ANN with the Cross-Entropy Loss for classification. However, they tend to be ineffective for OOD detection[2] because the Cross-Entropy Loss primarily optimizes for confident classification within known classes, leading models to produce high-confidence predictions even for OOD inputs. This occurs due to the softmax function, which normalizes outputs into a probability distribution, masking uncertainty and causing miscalibration. Additionally, cross-entropy is class-centric and does not consider whether an input is substantially different from the training distribution, often yielding low loss for OOD samples that resemble in-distribution data. Consequently, cross-entropy fails to adequately distinguish OOD data, necessitating specialized methods for reliable OOD detection.

We have added a comparison with deterministic methods for OOD detection like Maximum Predicted Softmax Probability[3], MaxLogit[4] and Energy-based ones[39] in the Extended Data Fig. 7. The inherent randomness of amplified spontaneous emission can greatly improve the security of communication systems, for example using the ASE as a random number generator for one-time pad encryption. In addition, ASE might be used in combination with coherent light sources to detect eavesdroppers in optical communication channels[5].

We have thus added a paragraph to the manuscript explaining the difference between uncertainty estimations with BNNs and deterministic ANNs.

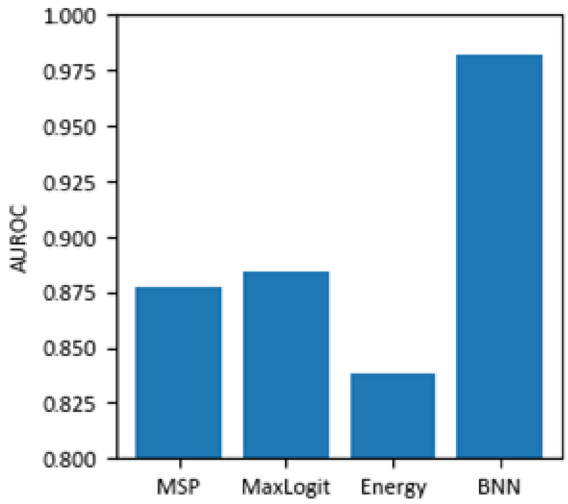

Figure: AUROC for OOD detection for different deterministic methods in comparison to the BNN

4. Limit to scalability explained in the discussion. What limits the number of channels that can be used for the same wavelength channel? Can you explain the tradeoff between coherence time, optical filter bandwidth and sampling rate?

Our Response:

Since the superposition of two independent chaotic light sources with identical spectra behaves as single chaotic light source with the same spectrum, the number of input channels to the photonic crossbar using the same wavelength channel can in principle be arbitrarily large. Practical limitations would, however, arise from the physical size of the photonic crossbar array, as well as fabrication tolerances and yields. Indeed, those three quantities are strongly connected and need to be chosen carefully for an optimal performance. The optical coherence time is linked to the spectral power density (and thus the filter bandwidth) by a Fourier transform, or more precisely via the Wiener–Khinchin theorem. As the coherence time quantifies the temporal correlation of the electric field / optical power, the time between two samples must be large in comparison to the coherence time. For example, in our case of a 200 GHz filter, the intensity / photodetector correlations drop to one (not zero, as the value is strictly positive) on a time scale of approximately 50 ps. Thus, we choose a time between two samples of 56.8 ps resulting in a sampling rate of 17.6 GS/s.

We have extended the paragraph about scaling in the main manuscript.

Minor Questions

- In Extended Data Fig. 3, what is meant by signal to noise ratio? How is it measured? The result is not intuitive to me. Also, how is the optical bandwidth experimentally manipulated as shown in Extended Data Fig. 3c? Is this just a simulation?

Our Response:

The Extended Data Fig. 3 is simulated according to Eq.S.1 of the supplementary materials to investigate the behaviour for an arbitrary optical bandwidth. This would indeed be really challenging to perform experimentally, as the Reviewer rightfully pointed out. The shown quantity is the ratio between the mean of the voltage traces shown in Extended Data Fig. 3b (referred to as signal) and the standard deviation of the voltage trace (referred to as noise). If the electronic bandwidth is larger than the optical one, the fluctuations are fully resolved, and the SNR is 1. For larger optical bandwidth, a part of the intensity fluctuations is averaged out by the detector and the SNR increases.

We have expanded the caption of the Figure to clarify this.

- In page 7, line 210, the authors write “In contrast, the standard deviation is not linearly decreased because the electronic ground noise of the readout circuit is independent from the optical intensity.” What is the source of electronic ground noise? Is this an intended behavior? In other words, does this scheme rely on the electronic ground noise (thermal noise?) to decouple mean from standard deviation? I thought the main mechanism was the chaotic light interference as explained in Eq. S3. Please clarify.

Our Response:

The reviewer is correct that the optical noise is the main mechanism for the probabilistic computing. Indeed, no electronic ground noise at all would drastically simplify the modelling and also improve the performance of the system, as we cannot modulate the ground noise in contrast the photonic one. The ground noise mainly arises from the thermal noise of the detector (and the transimpedance amplifier) converting the optical power to a voltage signal. However, it is important to include this noise term in the model to fully explain the measurement data. We thank the reviewers for pointing out the not precise formulation. We have modified it in the revised manuscript.

- On page 8, line 226, the authors write "As in the deterministic (convolutional) counterpart 35, we encode the convolution kernel weights in the GST cells and encode the image pixel values in the mean input intensities." In convolutional kernels, the kernel component for different colors/channels is different. In this architecture, the GST-based weights cause the kernel component to be color-insensitive. This draws a fundamental limitation of the convolution capability. Can you clarify what it would take to use this hardware for multi-channel/multi-dimensional kernels?

Our Response:

For the chosen Le-Net 5 model the average pooling layers only pool within one feature map and do not map N feature maps / input channels to M feature maps / output channels. Therefore, the crossbar is sufficient for this specific pooling operation. For arbitrary cases this is of course not the case, as the reviewer correctly pointed out. In that case, each "column" of the crossbar array would correspond to one output channel and the number of inputs to crossbar must be # kernel elements x # input channels. Therefore, the same architecture can be used, however, a larger crossbar would be required.

We have expanded to scaling paragraph in the discussion of the manuscript to applications with arbitrary convolution kernels.

- Typo in Fig. 5a "kernal"

- Typo in Supp. Fig. 6a "probabilsitc"

- Typo in Bayesian Neural Network tooling section, "hyperparamter"

Our Response:

We thank the reviewer for pointing out those typos and we corrected them.

We hope that the additional clarifications and additions to the manuscript have addressed the concerns of the referee and hope that she/he will support publication of our results.

Reviewer #2:

- Intro

In recent years, analog computing has experienced a resurgence thanks to the latest achievements in deep learning (DL). This research topic is of great interest, with efforts being made by both academic researchers and prominent companies such as IBM, NVIDIA, and Google. With the enormous potential of DL no longer under discussion, the effort is now on finding new ways to deploy these AI models efficiently in terms of computing speed, throughput, and, most importantly, power consumption. Analog photonics, in particular, has already demonstrated its ability to implement a plethora of DL models, from fully connected networks, to spiking neural networks and even reservoir computing.

Analog devices for AI fall into two main categories: (I) neuromorphic processors, which emulate the architecture and operating principles of DL, and (II) AI co-processor accelerators, which aim to improve a specific part of the DL workflow. The latter tipically aim to improve matrix multiplications, as they constitute the most demanding workload in DL inference.

In this paper, the authors report a method for developing a neuromorphic processor capable of implementing a Bayesian neural network (BNN). Their device relies on amplified spontaneous emission to implement a chaotic light (an ideal entropy source). Having a broad optical bandwidth, this chaotic light can be used in a photonic crossbar array in wavelength division multiplexing (WDM). The crossbars are based on phase-change materials acting on the amplitude of light as tuneable weighting elements.

Our Response:

We thank the referee for taking the time to thoroughly review our submission.

- Comments on the manuscript

In the manuscript the authors propose an interesting method to implement a Bayesian neural network (BNN) with photonics. Instead of trying to implement a more “standard” network such as a multilayer perceptron, where noise limits the scalability and resolution of photonic implementations, they focus on an architecture where photonic noise is a resource rather than a burden. From another perspective, instead of trying to somehow replicate digital electronics, their device aims to enable a novel functionality (the implementation of a BNN) that is beyond the reach of its electronics counterpart. The BNN is implemented in photonics through a chaotic light source, electro absorption modulators (EAM) used to impress inputs in time amplitude modulation, a photonic crossbar array with phase change materials (PCM) as the WDM weighting matrix, and photodiodes attached to wavelength demultiplexers.

The authors provide a convincing discussion of why relying on WDM photonics for probabilistic computing overcomes the shortcomings of electronic counterparts (high speed, broadband optical sources, avoidance of sequential sampling, use of a single source).The authors provide a convincing discussion regarding why relying on WDM photonics for probabilistic computing overcomes the shortcomings in the electronic counterparts (high speed, broadband optical sources, avoidance of sequential sampling, use of a single source).

The manuscript provides sufficient information on the probabilistic encoding of inputs (i.e., how both the mean and the variance of input distributions can be modulated) and how the PCMs are used within the photonic crossbar to perform multiplications acting on the amplitude of optical signals.

The combination of the main and supplementary material provides sufficient information for the reproducibility of the work. Images are well composed, with appropriate captions, and of a sufficiently high resolution. The English is generally good, with a few typos (e.g., "in fast and efficient way" at page 4).

Our Response:

We thank the reviewer for affirming the novelty of our work and his/her detailed comments to further improve the manuscript. We modified it as outlined in the following response to all comments.

However, the manuscript has some shortcomings that should be addressed to improve its quality, and the understanding of the potential impact of the presented research.

The manuscript lacks a state-of-the-art comparison with other photonic probabilistic processors. Some references to some other works are provided, but it lacks a proper comparison that would allow to assess the degree of novelty introduced by this work and its potential impact.

Our Response:

We thank the referee for the suggestion to provide a more extended comparison with other probabilistic processors to improve the quality of the manuscript.

In general, the research can be divided into two different directions, fully probabilistic architectures like ours and combinations of photonic and deterministic hardware. Combined systems could consist of FPGAs computing the matrix vector multiplications and a photonic random number generation at the activation function, realizing a binary probabilistic activation function via a bistable optical parametric oscillator as shown in Ref[1]. There, the authors realized a sampling rate of 10 kS/S for the system and envision sampling rates in the order of 1 GS/s for future realizations. Similarly, photonics may be used to compute MVMs and be linked with a digital activation function and pseudo random generator to solve combinatorial problems[6]. However, in ref 6 the authors do explain the concepts in detail but only simulate it on an FPGA. Further work[7] proposes an all-optical solution mixing coherent light with chaotic light. Here, the authors characterize a single probabilistic synapse and envision the full processor, however, suffer from a drastically more complicated architecture including multiple rings aligned to each other and a mixture of several coherent and chaotic light sources that need to be properly aligned with respect to each other.

In this sense, to the best of our knowledge our photonic probabilistic processor is the first all optical implementation. We included the discussion and the additional references in the main manuscript.

A discussion of the scalability and possible integration of such a device would improve the quality of the paper. In this regard, the authors state "[…] employing chaotic light as a carrier leads to interesting scaling properties." but this is developed in a non-quantitative way with "Note that in our experiments the number of parallel samples was mainly limited by the number of input channels of the oscilloscope […]". To how many input channels could the structure be scaled? Presumably, the photonic crossbar array may be impractical to be implemented when increasing the number of inputs, since its dimension scales quadratically. Moreover, do the authors envisage a way to integrate the device? A semiconductor optical amplifier can be used as the chaotic light source, but programming the PCMs may become a challenge if everything is integrated (?).

Our Response:

For the default deterministic crossbar architecture[8] the number of different wavelengths in the systems is given by the number of inputs to the crossbar (without parallel computing via WDM). Each of the inputs needs to be properly detuned in order to avoid measurable interference effects. In contrast, we can employ the same frequency range for all inputs, thereby drastically simplifying the wavelength management. The reviewer is correct, that photonic crossbar arrays in general suffer from scaling problems in comparison to their electronic counterparts[9] due to the larger size of the photonic crossbar array. For scaling, switching from the SiN to the SOI platform will be beneficial, as tighter bend radii (in the order of 5 µm) can be realized and thus the size of the matrix cell reduced to about 30 µm x 30 µm[8]. In addition, SOI enables the use of compact PIN heaters that could be employed for PCM switching[10]. In addition, the integration of Indium Phosphide as a gain medium is also possible[11,12].

While the first implementation of a novel device is almost always interesting in itself, a section discussing its shortcomings and possible turnarounds would be impactful. The paper has the potential to become a reference for the field of probabilistic photonic computing.

Our Response:

We thank the reviewer for his appreciation of our work. We included a new paragraph in the discussion, especially focussing on future scaling, challenges and the potential of our architecture.

Regarding the speed of the device, the authors report an overall sampling rate of about 70 GSa/s. However, while the sampling rate is given, the authors do not outline the speed of their processor in terms of throughput and actual input generation rate (sequential pulses are used to shape input distributions). For example, a processor based on a Mach-Zehnder mesh has a throughput limited by the light crossing time of the device. As for how a BNN works, the output is a series of statistics. It is not clear from the paper whether extracting such statistics requires only a forward pass or not.

Our Response:

There are several characteristics contributing to the final speed and throughput of the processor. As the reviewer pointed out, the size of the device limits the minimal latency of the architecture. With a group velocity in SiN waveguides at 1550 nm of about 150.000 km/s and a crossbar array with a mm to cm scale, the minimal latency time is on the order of 6 to 60 ps. As 9 subsequent pulses are needed to encode the distribution and the symbol rate for one channel is 17.6 GBaud, the total latency time for the full probabilistic computation is on the order of 550 ps. Parallel sampling does not decrease the latency of the computation but increases the throughput, as several samples are required to gather the output statistics of the BNN.

The optimal number of samples is usually very problem-specific, depending on the neural architecture as well as the problem-at-hand. For the MNIST problem, we compute the number of true and false positives in the OOD detection problem for different thresholds in the Mutual Information score. Subsequently, we can compute the AUROC (Area Under Receiver Operating Characteristic). The research community generally uses this metric to gauge the quality of the OOD test. An AUROC score of 1 would indicate that the OOD classifier can perfectly detect OOD samples. In general, a good rule of thumb is to use more than 10s of samples for a thorough analysis of an algorithm. To be on the safe side, in the experiments here we used at least 100 samples.

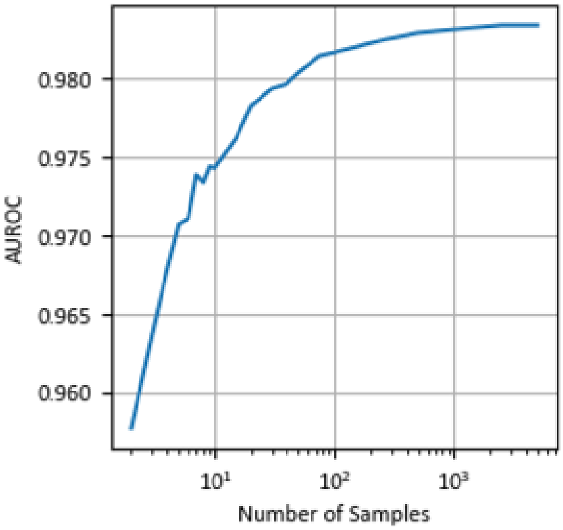

Figure: AUROC for OOD detection depending on the number of samples.

Considering the use of $s = 100$ samples and $w = 4$ wavelength channels, we perform the same operation s/w times after each other, resulting in a total latency time of 13.75 ns. This time could be reduced by using more parallel channels as only a fraction of the C-Band, where the Erbium doped fiber ASE source emits, is used.

We have included the graph in the Extended Data file and a further discussion in the Supplementary information.

Furthermore, the photonic probabilistic processor is only used in 2 out of 7 layers within the developed BNN (other layers are deterministic). How does this affect the computation of the final layers of the BNN, which are run on a digital computer? Do these need to be run several times as the output of the probabilistic layers are distributions? The authors could reasonably respond to this comment by saying that the answer should be obvious if you know how BNN works. However, providing some insight into this would allow the paper to be understood by a wider audience.

Our Response:

We thank the reviewer for the suggestion to provide a more detailed explanation to make the article easier accessible to a wider audience.

The subsequent layers on digital HW indeed need to be evaluated several times. In general, our approach is a partially-stochastic. The question how many and which layers should be probabilistic is an open discussion in the research community[13]. While first findings show that NNs do not need to be fully stochastic, particular details, such as how many layers and which layers, seem to highly depend on the task at hand. Long term, a hybrid system of photonic probabilistic layers for high-speed convolution processing and sampling in combination with deterministic electronic cores is especially intriguing, combining the advantages of photonics for probabilistic computing with the scaling and memory advantage of electronic systems.

We have included a discussion on future scaling and challenges in the discussion.

- Summary

The authors propose a novel and interesting solution for implementing a probabilistic photonic processor. The potential of this work may be significant, but a proper comparison with the state of the art is required to assess its actual degree of novelty. Addressing the issues raised in this review should

improve the quality of the work and potentially make it suitable for publication in Nature Communications.

Our Response:

We thank the reviewer for outlining the significance of our work and hope that with the additional clarifications and comparisons in the manuscript she/he will be supportive of publication of our results.

References:

1. Choi, S. *et al.* Photonic probabilistic machine learning using quantum vacuum noise. 1–8 (2024).
2. Nguyen, A., Yosinski, J. & Clune, J. Deep neural networks are easily fooled: High confidence predictions for unrecognizable images. *Proc. IEEE Comput. Soc. Conf. Comput. Vis. Pattern Recognit.* **07-12-June**, 427–436 (2015).
3. Hendrycks, D. & Gimpel, K. A baseline for detecting misclassified and out-of-distribution examples in neural networks. *5th Int. Conf. Learn. Represent. ICLR 2017 - Conf. Track Proc.* 1–12 (2017).
4. Hendrycks, D. *et al.* Scaling Out-of-Distribution Detection for Real-World Settings. (2022).
5. Zhu, H., Liu, Z., Chen, S., Xu, X. & Li, F. Optical stealth communication based on quantum noise stream ciphered amplified spontaneous emission light. *Opt. Express* **31**, 3595 (2023).
6. Roques-Carmes, C. *et al.* Heuristic recurrent algorithms for photonic Ising machines. *Nat. Commun.* **11**, (2020).
7. Wu, C., Yang, X., Chen, Y. & Li, M. Photonic Bayesian Neural Network Using Programmed Optical Noises. *IEEE J. Sel. Top. Quantum Electron.* **29**, (2023).
8. Feldmann, J. *et al.* Parallel convolutional processing using an integrated photonic tensor core. *Nature* **589**, 52–58 (2021).
9. Gallo, M. Le *et al.* A 64-core mixed-signal in-memory compute chip based on phase-change memory for deep neural network inference. *Nat. Electron.* **6**, (2023).
10. Erickson, J. R. *et al.* Comparing the thermal performance and endurance of resistive and PIN silicon microheaters for phase-change photonic applications. *Opt. Mater. Express* **13**, 1677 (2023).
11. Yan, Z. *et al.* A monolithic InP/SOI platform for integrated photonics. *Light Sci. Appl.* **10**, (2021).
12. Xiang, C. *et al.* 3D integration enables ultralow-noise isolator-free lasers in silicon photonics. *Nature* **620**, 78–85 (2023).
13. Sharma, M., Farquhar, S., Nalisnick, E. & Rainforth, T. Do Bayesian Neural Networks Need To Be Fully Stochastic? *Proc. Mach. Learn. Res.* **206**, 7694–7722 (2023).

Response Letter Probabilistic Photonic Computing with Chaotic Light

Frank Brückerhoff-Plückelmann, Hendrik Borras, Bernhard Klein, Akhil Varri, Marlon Becker, Jelle Dijkstra, Martin Brückerhoff, C. David Wright, Martin Salinga, Harish Bhaskaran, Benjamin Risse, Holger Fröning, Wolfram Pernice

Response to reviewers

We thank both referees for approving the manuscript and their final comments. We made a minor change to the main text, as described below.

Reviewer #1:

In a previous review, I had recommended the editor to accept this for publication after providing minor revisions. In this iteration, the authors have satisfactorily addressed my revision suggestions. I appreciate the insight into how deterministic approaches are inferior in rejecting OOD samples. I still suspect that relatively small optimizations in deterministic hardware or software algorithms would be sufficient to improve probabilistic neural networks performance. As this field develops, it would be great to gain insight into what fundamentally can only be achieved via a physical entropy source and hardware-assisted PDF evaluation. It is understood that this is, however, outside the scope of this article

Our Response:

We thank the reviewer for the positive evaluation of our paper. We fully agree that benchmarking the photonic probabilistic processor with deterministic hardware for real world applications will finally determine the overall usefulness and looking forward to explore this field in the future.

Reviewer #2:

The authors have carefully addressed all the issues raised in my previous review. The overall quality of the paper has improved significantly. In my opinion, the paper is more reader-friendly. It has the potential to have a good impact, which is paramount.

I confirm all my positive comments on the previous review and hope to see more efforts from the scientific community in photonic probabilistic computing in the future. As I said, I particularly appreciated the use of optical noise as a resource rather than a burden.

I think the paper is now ready for publication in Nature Communications.

I thank the authors for all their responses. As a side, scientific consideration, I must disagree with a statement in the authors' response regarding scalability. The authors say that "photonic crossbar arrays in general suffer from scaling problems compared to their electronic counterparts". If we consider memristor crossbar arrays as the electronic counterpart, their scalability is also severely limited by parasitic effects. In this case, photonics has a better perspective, as in principle even long interconnections can be supported. The technological advances in fabrication processes will tell us how much we can push in scalability.

Our Response:

We thank the referee for the final approval of our paper. Indeed, when referring to “scaling problems” we mainly thought of the lower memory density in photonics, which thus likely requires larger circuit areas than the equivalent electronic counterparts, and not the problems arising from fabricating larger circuits, which will likely be more feasible in photonics due to the better interconnects as the reviewer pointed out. Advancing to the limits given by the fabrication processes will indeed be very interesting for the general benchmarking of photonic processors.
